# Application of highly sensitive saturation labeling to the analysis of differential protein expression in infected ticks from limited samples

**DOI:** 10.1186/1477-5956-8-43

**Published:** 2010-08-12

**Authors:** Margarita Villar, Alessandra Torina, Yolanda Nuñez, Zorica Zivkovic, Anabel Marina, Angela Alongi, Salvatore Scimeca, Giuseppa La Barbera, Santo Caracappa, Jesús Vázquez, José de la Fuente

**Affiliations:** 1 Instituto de Investigación en Recursos Cinegéticos IREC (CSIC-UCLM-JCCM), Ronda de Toledo s/n, 13005 Ciudad Real, Spain; 2 Intituto Zooprofilattico Sperimentale della Sicilia, Via G. Marinuzzi n°3, 90129 Palermo, Sicily, Italy; 3 Centro de Biología Molecular "Severo Ochoa" (CSIC-UAM), 28049 Cantoblanco, Madrid, Spain; 4 Utrecht Centre for Tick-borne Diseases (UCTD), Department of Infectious Diseases and Immunology, Faculty of Veterinary Medicine, Utrecht University, Yalelaan 1, 3584CL, Utrecht, The Netherlands; 5 Department of Veterinary Pathobiology, Center for Veterinary Health Sciences, Oklahoma State University, Stillwater, OK 74078, USA

## Abstract

**Background:**

Ticks are vectors of pathogens that affect human and animal health worldwide. Proteomics and genomics studies of infected ticks are required to understand tick-pathogen interactions and identify potential vaccine antigens to control pathogen transmission. One of the limitations for proteomics research in ticks is the amount of protein that can be obtained from these organisms. In the work reported here, individual naturally-infected and uninfected *Rhipicephalus *spp. ticks were processed using a method that permits simultaneous extraction of DNA, RNA and proteins. This approach allowed using DNA to determine pathogen infection, protein for proteomics studies and RNA to characterize mRNA levels for some of the differentially expressed proteins. Differential protein expression in response to natural infection with different pathogens was characterized by two-dimensional (2-D) differential in gel electrophoresis (DIGE) saturation labeling in combination with mass spectrometry analysis. To our knowledge, this is the first report of the application of DIGE saturation labeling to study tick proteins.

**Results:**

Questing and feeding *Rhipicephalus *spp. adult ticks were collected in 27 farms located in different Sicilian regions. From 300 collected ticks, only 16 were found to be infected: *R. sanguineus *with *Rickettsia conorii *and *Ehrlichia canis*; *R. bursa *with *Theileria annulata*; and *R. turanicus *with *Anaplasma ovis*. The proteomic analysis conducted from a limited amount of proteins allowed the identification of host, pathogen and tick proteins differentially expressed as a consequence of infection.

**Conclusion:**

These results showed that DIGE saturation labeling is a powerful technology for proteomics studies in small number of ticks and provided new information about the effect of pathogen infection in ticks.

## Background

Ticks are ectoparasites of wild and domestic animals and humans, and are considered to be the most important arthropod vector of pathogens in some regions of the world [1]. In particular, *Rhipicephalus *spp. ticks transmit pathogens of the genera *Anaplasma*, *Ehrlichia*, *Rickettsia*, *Babesia *and *Theileria *that impact both human and animal health [1,2]. The ticks and the pathogens that they transmit have co-evolved molecular interactions involving genetic traits of both the tick and the pathogen that mediate their development and survival [3-5].

Due to complexities of working with ticks and despite great advances in proteomics technologies during the last decades, proteomics studies to characterize protein expression in ticks are difficult to conduct [5-17]. Most of these studies have focused on the *sialome *(salivary gland secretory proteome) analysis of ticks [6,7,9,13-15] and the analysis of host-tick-pathogen interactions in an attempt to identify potential candidates for vaccine development against vector-borne diseases [5,8,10-12,16,17].

One of the limitations for proteomics research in ticks is the amount of protein that can be obtained from these organisms. The saturation difference in gel electrophoresis (DIGE) technology recently developed has emerged as a useful method for protein analysis from scarce amounts of protein [18,19]. Protein labeling in saturation DIGE is based on dyes that have a maleimide reactive group that form a covalent bond with the thiol group of cysteine residues via a thioether linkage, whereas the reactive group of dyes for the minimal DIGE labeling is a NHS ester that react with the epsilon amino group of lysine residues in proteins via an amide linkage. Moreover, as its name suggests, in saturation labeling the dyes are added to the protein under such conditions that all available cysteine residues of a protein are labeled, in contrast to minimal DIGE labeling technology in which only 1-3% of lysine residues are labeled [20]. This new strategy results in a strongly enhanced sensitivity when compared to minimal DIGE methodology, with a detection limit established at 0.1 ng of albumin [18]. Thereby, saturation DIGE labeling appears to be a highly suitable strategy for proteome studies using small amounts of protein samples providing results from sample quantities 10-fold lower than those required to carry out the minimal labeling approach.

In the work reported herein, individual infected and uninfected *Rhipicephalus *spp. ticks were processed using a method that permits simultaneous extraction of DNA, RNA and protein to characterize differential protein expression in response to natural infection with different pathogens by two-dimensional DIGE saturation labeling in combination with mass spectrometry analysis. The results showed that DIGE saturation labeling is a powerful technology for proteomics studies in small number of ticks, even when proteins are extracted by methods that allow simultaneous analysis of DNA and RNA samples. To our knowledge, this is the first report of the application of DIGE saturation labeling to study tick proteins.

## Material and methods

### Ticks and DNA/RNA/protein extraction

Questing and feeding *Rhipicephalus *spp. adult female ticks were collected in 27 farms located in different Sicilian regions (Palermo, Enna, Messina, Siracusa and Trapani). A total of 300 ticks were collected and analyzed for this study. Of them, 12 were questing ticks and 288 were fully engorged ticks collected from sheep, goats or dogs. These ticks were collected from adult animals living in pathogen endemic areas, thus likely to have chronic infections. Ticks were identified using morphological keys for the Italian Ixodidae [21]. The ticks were incubated for three days in the laboratory prior to dissection and RNA/DNA/protein extraction. Individual ticks were dissected and whole internal organs extracted and used for DNA, RNA and protein extraction using TriReagent (Sigma, St. Louis, MO, USA) following manufacturers recommendations. Animal experiments were conducted with the approval and supervision of the Intituto Zooprofilattico Sperimentale della Sicilia Institutional Animal Care and Use Committee (project IZS SI 10-06).

### Identification of pathogen infection in naturally infected ticks

The DNA was resuspended in sterile distilled water and stored at -20°C until used. For the initial screening, PCR analyses for *Anaplasma*, *Ehrlichia *and *Rickettsia *spp. were performed as described previously [22] with 1 μl (0.1-10 ng) DNA using 10 pmol of each primer and the Ready-To-Go PCR beads (Amersham, Piscataway, NJ, USA). Reactions were performed in an automated DNA thermal cycler for 35 cycles. PCR products were electrophoresed on 1% agarose gels to check the size of amplified fragments by comparison to a DNA molecular weight marker (1 Kb DNA Ladder, Promega, Madison, WI, USA). Control reactions were done without the addition of DNA to the reaction to rule out contaminations during PCR. Reverse line blot (RLB) was used for detection of *Babesia *and *Theileria *spp. as described previously [23].

To confirm pathogens in ticks, PCR and sequence analysis of cloned amplicons were performed for *Anaplasma*, *Ehrlichia *and *Rickettsia *spp. Amplified fragments were resin purified (Promega), cloned into pGEM-T vector (Promega) and sequenced in an accredited service laboratory (BaseClear, Leiden, The Netherlands) using vector specific primers. The BLAST tool was used to search the NCBI databases in order to identify sequences reported previously with identity to sequences obtained herein. Gene sequences were deposited in the GenBank with accession numbers GQ857075-GQ857078 . RLB was used to confirm *T. annulata *infection.

### Two-Dimensional Difference in Gel Electrophoresis (2-D DIGE)

Following protein extraction with TriReagent (Sigma), protein samples were purified using a 2-D Clean-Up Kit (GE Healthcare, Madrid, Spain) according to the manufacturer's instructions to remove any contaminant substances that could interfere with the 2-D DIGE procedure. The protein pellet was resuspended in 25 μl of 2-D lysis buffer (7 M urea, 2 M thiourea, 4% w/v CHAPS, 25 mM Tris-HCl, pH 8.0) and protein concentration was determined using the 2D-Quant Kit (GE Healthcare).

In order to reduce variance from individual-to-individual variation and to obtain enough protein quantity to perform the experiment, protein samples from the same experimental group were pooled. Consequently, a total of 8 different samples were analyzed by 2D-DIGE, four infected with *R. conorii*, *E. canis*, *T. annulata *or *A. ovis *and their respective uninfected controls of the same tick species and collected from the same type of host or off-host.

CyDye DIGE fluor labeling kit for scarce protein samples (GE Healthcare) was used to label tick proteins according to the manufacturer's protocol. Briefly, for cysteine reduction before labeling, 5 μg of protein of each sample were incubated with 2 nmol Tris (2carboxyethyl) phosphine hydrochloride (TCEP; Sigma) at 37°C for 1 hour in the dark and, for labeling, 4 nmol of Cy5 dye in 2 μl of anhydrous DMF (Sigma) were added and the samples were incubated at 37°C for 30 min in the dark. For internal standardization, a pool of equal amounts of all samples (5 μg per sample) was created and labeled with Cy3 dye with the same procedure but scaling adjusting the quantities of reagents according to the amount of protein (40 μg). The reaction was quenched by adding and equal volume of 2 × sample buffer (7 M urea, 2 M thiourea, 4% w/v CHAPS, 1% v/v IPG buffer pH 3-11, 0.2% w/v DTT). Before 2-D separation, 5 μg of the Cy3-pool was mixed with 5 μg of each sample.

For the first dimension, 24-cm 3-11 NL pH range IPG strips were rehydrated overnight in 450 μL of DeStreak Rehydration Solution (GE Healthcare) supplemented with 0.5% IPG buffer pH 3-11 (GE Healthcare) using a reswelling tray. IEF was performed at 20°C using an Ettan IPGphor 3 (GE Healthcare). Samples were applied using anodic cup loading and the isoeletrofocusing was carried out using the following conditions: 300 V for 3 h, 300-1000 V for 6 h, 1000-10000 V for 3 h, 10000 V for 3 h and 500 V for 3 h. Prior to second dimension, focused IPG strips were incubated for 10 min equilibration buffer containing 50 mM Tris-HCl pH 8.8, 6 M urea, 30% v/v glycerol, 2% w/v SDS, 0.5% w/v DTT and traces of bromophenol blue. Equilibrated IPG strips were placed onto 12% homogeneous SDS-polyacrylamide gels casted in low fluorescence glass plates using an Ettan-DALT Six System (GE Healthcare). Electrophoresis was carried out at 20°C and 0.5 W/gel for 30 min followed by a second step at 15 W/gel for 4 hours.

### Image acquisition and data analysis

Proteins were visualized using an Ettan DIGE Imager (GE Healthcare) following the manufacturer's instructions. Image analysis was performed with DeCyder 2 D Software, version 7.0 (GE Healthcare). Sixteen images were considered for the analysis, 8 corresponded to the different samples labeled with Cy5 and 8 corresponded to sample pool labeled with Cy3 and acquired individually with each gel. Spot co-detection, normalization of each spot against the corresponding value of the internal pool and volume ratios calculation were carried out using Differential In-Gel Analysis (DIA) module. In the Biological Variation Analysis (BVA) module, the 16 spot maps were distributed in 9 groups, that is, standard, and the 8 different samples (4 controls and 4 infected) and the standard image most representative with average quality was assigned as the master image. After matching of images, average ratios between groups were calculated. Protein spots with 5-fold as threshold in the average ratio were considered as differentially expressed between the samples under comparison.

### Selection and preparation of protein samples for mass spectrometry

For preparative gels, equal amounts of all samples were pooled. Due to sample limitation, only 8 μg of each sample were mixed obtaining 64 μg of total proteins. 2-D electrophoresis was carried out in the same conditions as described above for CyDye labeled samples, but in this case, after second dimension, the gel was stained with Sypro Ruby (Molecular Probes, Invitrogen, Eugene, OR, USA) following the protocol recommended by the manufacturer. Proteins were visualized by fluorescence using an Ettan DIGE Imager (GE Healthcare) selecting 100 μm as pixel size and channel Sypro Ruby 1 with 0.4 of exposure to acquire the gel image. The gel was matched automatically in the BVA module of DeCyder software with the DIGE images. Of all spots matching with this gel, only those spots that were identified as differentially expressed and appeared at least in two of the four tick-pathogen groups under comparison were selected for mass spectrometry analysis. The 2-D electrophoresis stained gel was washed twice for 10 min with distilled water. Selected protein spots were visualized with an UV benchtop transilluminator (UVP, Cambridge, UK), manually excised from the gels, dehydrated with acetonitrile and vacuum-dried (Savant Speed Vac, mod SPD, 121 P, equipped with a vacuum pump OFP-400). After drying, spots were re-hydrated and digested *in situ *with trypsin (Promega) as described by Shevchenko et al. [24] with minor modifications. Stained protein gel spots were incubated in 50 mM NH_4_HCO_3 _with trypsin (5 ng/μl) for 1 hr in an ice-bath. The digestion buffer was removed and gels were covered again with 50 mM NH_4_HCO_3 _and incubated at 37°C for 12 hr. Whole supernatants were allowed to dry and then stored at 20°C until mass spectrometry analysis.

### Matrix-assisted laser desorption/ionization-time of flight mass spectrometry (MALDI-TOF MS) analysis

Peptide mass fingerprinting was conducted as previously described [25] using an Autoflex™(Bruker Daltonics, Bremen, Germany) mass spectrometer in a positive ion reflector mode employing 2, 5-dihydroxybenzoic acid as matrix and an AnchorChip™surface target (Bruker Daltonics). Peak identification and monoisotopic peptide mass assignation were performed automatically using Flexanalysis™software, version 2.2 (Bruker Daltonics). Database searches were performed using MASCOT http://matrixscience.com[26] against the NCBI non-redundant protein sequence database http://www.ncbi.nih.gov. The selected search parameters were as follows: tolerance of two missed cleavages, carbamidomethylation (Cys) and oxidation (Met) as fixed and variable modifications, respectively, and setting peptide tolerance to 100 ppm after close-external calibration. A significant MASCOT probability score (*p <*0.05) was considered as condition for successful protein identification.

### Reverse phase-liquid chromatography (RP-LC)-MS/MS analysis

When peptide mass fingerprinting failed to identify a spot, the protein digest was dried, resuspended in 7 ul of 0.1% formic acid and analyzed by RP-LC-MS/MS in a Surveyor HPLC system coupled to an ion trap Deca XP mass spectrometer (Thermo Fisher Scientific, Waltham, MA, USA). The peptides were separated by reverse phase chromatography using a 0.18 mm × 150 mm BioBasic C18 RP column (Thermo Fisher Scientific), operating at 1.8 μl/min. Peptides were eluted using a 50-min gradient from 5 to 40% solvent B (Solvent A: 0,1% formic acid in water, solvent B 0,1% formic acid, 80% acetonitrile in water). ESI ionization was done using a microspray "metal needle kit" (Thermo Fisher Scientific) interface. Peptides were detected in survey scans from 400 to 1600 amu (8 μscans), followed by three data-dependent MS/MS scans, using an isolation width of 3 amu, normalized collision energy of 30%, and dynamic exclusion, applied during 3-min periods.

Peptide identification from raw data was carried out using the SEQUEST algorithm (Bioworks Browser 3.2, Thermo Fisher Scientific) and the PHENYX 2.6 search engine (GENEBIO, Switzerland). Database search was performed against the Apicomplexa, α-proteobacteria and metazoa databases downloaded from the Protein Knowledgebase (UniProtKB) http://www.uniprot.org. The following constraints were used for the searches: tryptic cleavage after Arg and Lys, up to two missed cleavage sites, and tolerances of 2 Da for precursor ions and 0.8 Da for MS/MS fragment ions and the searches were performed allowing optional Met oxidation and fixed Cys carbamidomethylation.

If the SEQUEST and PHENYX searches did not yield any positive results, high-quality spectra that had not been assigned to any protein identification were selected and manual *de novo *interpretation was conducted. These results were confirmed with PEAKS Studio 4.5 software (Bioinformatics Solutions Inc., Waterloo, ON, Canada).

### Analysis of mRNA levels by real-time RT-PCR in naturally infected ticks

The genes encoding for differentially expressed unknown larval (Genbank accession number EF675686) and guanine nucleotide-binding (DQ066296) proteins were selected for mRNA analysis by real-time RT-PCR. mRNA levels were characterized in individual whole ticks naturally-infected with different pathogens using sequence-specific oligonucleotide primers (unknown larval protein, UNLP-F: 5'-TCATCCTCTGTGTGCTCGTC and UNLP-R: 5'-TCTCGAGGCAAGTGTCAATG; guanine nucleotide-binding protein, GNBP-F: 5'-GGGACTTGGAGGGCAAGAG and GNBP-R: 5'-ACACCTGCCAGACCCTGAT) as described previously [27]. In all cases, matching groups of uninfected tick samples were analyzed concurrently for comparison. Real-time RT-PCR was done using the QuantiTec SYBR Green RT-PCR kit (Qiagen, Valencia, CA, USA) and a Bio-Rad iQ5 thermal cycler (Hercules, CA, USA) following manufacturer's recommendations. mRNA levels were normalized against tick 16 S rRNA using the comparative Ct method [27].

## Results and discussion

In this work, a 2-D DIGE saturation labeling approach in combination with MS was used to characterize differential protein expression in *Rhipicephalus *spp. ticks naturally infected with different pathogens. Each particular tick species infected with a pathogen was compared with its respective uninfected control of the same tick species and collected from the same host or off-host. Individual infected and uninfected control ticks were processed using a method that permits simultaneous extraction of DNA, RNA and protein. This approach allowed using DNA to determine pathogen infection, protein for proteomics studies and RNA to characterize mRNA levels for some of the differentially expressed proteins.

### Analysis of pathogen infection in *Rhipicephalus *spp. ticks

After collection in different farms, *Rhipicephalus *spp. ticks were analyzed for *Anaplasma*, *Ehrlichia*, *Rickettsia*, and *Theileria *spp. infection by PCR and RLB. From 300 ticks collected, only 16 were found to be infected: *R. sanguineus *was infected with *R. conorii *and *E. canis*; *R. bursa *was infected with *T. annulata*; and *R. turanicus *was infected with *A. ovis *(Table 1). The number of infected *Rhipicephalus *spp. ticks was low (2-9 per group; Table 1), a finding that may be common in tick field studies depending on the prevalence of tick infestations and pathogen infection [22,28,29]. Uninfected ticks were confirmed to be negative for all pathogens analyzed.

**Table 1:** Rhipicephalus spp. ticks naturally infected with Rickettsia, Ehrlichia, Theileria or Anaplasma species.

Tick species	Collection	Pathogen infection	N ^a)^	Total proteins extracted (μg) b)
*R. sanguineus*	questing	*R. conorii*	3	57.9

*R. sanguineus*	dog	*E. canis*	2	40.1

*R. bursa*	sheep	*T. annulata*	9	92.2

*R. turanicus*	sheep	*A. ovis*	2	18.5

Questing and feeding *Rhipicephalus *spp. adult female ticks were collected in Sicilian farms and analyzed for pathogen infection by PCR or RLB. To define pathogen species infecting ticks, PCR and sequence analysis of cloned amplicons were performed for *Anaplasma*, *Ehrlichia *and *Rickettsia *spp. For *Theileria *spp., RLB results were confirmed at the species level. For proteomics analysis, sex and collection-matching uninfected controls were used. Uninfected ticks were negative for all pathogens analyzed.

^a) ^Number of ticks comprising the sample pool for 2-DE.

^b) ^After protein extraction with TriReagent (Sigma), interfering components for 2D-DIGE experiments were removed by using 2D-Clean up Kit (GE Healthcare) and protein concentration was determined using the 2D-Quant Kit (GE Healthcare). For analysis, protein samples were pooled and table shows the total quantity obtained for each group.

### 2-D DIGE analysis of tick protein profiles

**Figure 1:**
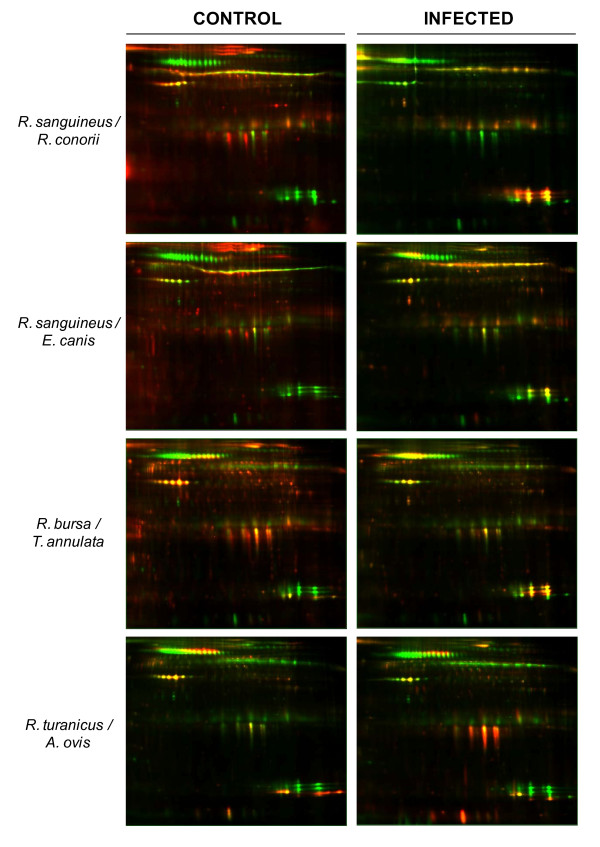
**DIGE overlay images of proteins from infected and uninfected *Rhipicephalus *spp.ticks**. Pooled internal standard proteome is represented in green (Cy3) and specific proteins for each sample are represented in red (Cy5).

Proteins obtained from the four different groups of infected *Rhipicephalus *spp. ticks and their respective uninfected controls were labeled with Cy5 saturation dye while the pooled internal standard was labeled with Cy3 saturation dye. Saturation labeling technology only employs two fluorochromes and it is not possible to include control and infected samples within the same gel, as in case of minimum labeling approach. Here, each sample mixed with the pooled internal standard was run separately in a gel. Thus, as shown in figure 1, this study comprised eight 2-D gels representing each individual group of samples. Two experiments were conducted with similar results.

**Figure 2:**
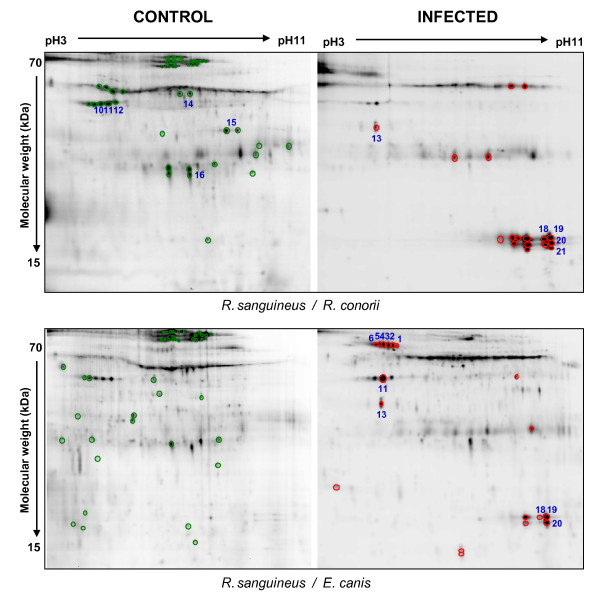
**Specific Cy5-labeled protein images of infected and uninfected *Rhipicephalus sanguineus *ticks**. Proteins that were differentially expressed with an average ratio of ± 5-fold are circled. Green and red circles represent proteins that were under-expressed or over-expressed after infection, respectively. Blue numbers indicate protein spots in Table 3 and figure 4 that were analyzed by MS.

**Figure 3:**
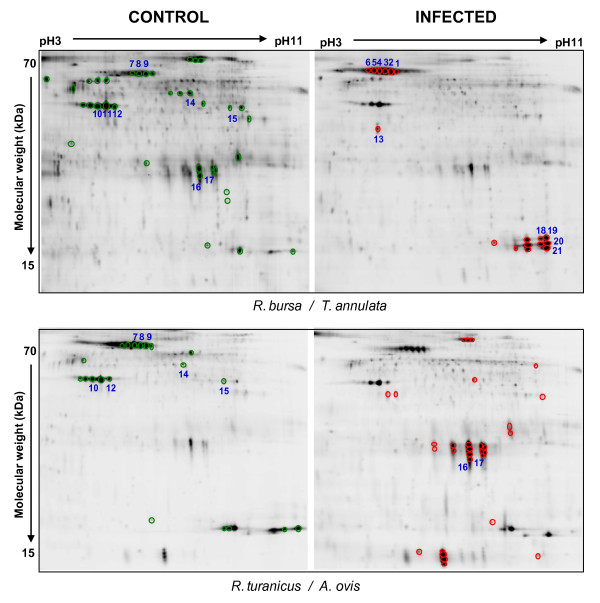
**Specific Cy5-labeled protein images of infected and uninfected *Rhipicephalus bursa and Rhipicephalus turanicus *ticks**. Proteins that were differentially expressed with an average ratio of ± 5-fold are circled. Green and red circles represent proteins that were under-expressed or over-expressed after infection, respectively. Blue numbers indicate protein spots in Table 3 and figure 4 that were analyzed by MS.

Evaluation of protein patterns of 16 spot maps obtained were performed with the DeCyder software. Between 1853 and 2484 spots were automatically detected, of which, an average of 1365 spots (SD = 63) were matched with the master gel. The group to group comparisons between control and infected spot maps were done and the differences obtained were considered significant when the calculated average ratio (infected *vs*. control samples) showed a value lower than -5 or higher than +5 (Figure 2 and 3; Table 2). Due to the limitation in protein sample quantity obtained from each tick group, it was necessary to mix samples from the same group. Pooled protein samples have the advantage of reducing variance from individual-to-individual variation, but it required the application of restrictive selection criteria due to the absence of statistical analysis. Tick proteins under-expressed after infection accounted for around 70% of differentially expressed proteins in all groups analyzed except in *R. turanicus*/*A. ovis *where 60% of differentially expressed proteins were over-expressed in infected ticks (Table 2).

**Table 2:** Summary of differentially expressed proteins in the comparative analysis of *Rhipicephalus *spp. adult female ticks naturally infected with different pathogens.

Tick/pathogen	Total numberof differentially expressed protein spots	Under expressedin infected ticks	Over expressedin infected ticks
*R. sanguineus/R. conorii*	65	48	17

*R. sanguineus/E. canis*	59	41	18

*R. bursa/T. annulata*	54	37	17

*R. turanicus/A. ovis*	50	20	30

Ticks infected with different pathogens were compared with their respective uninfected controls and the proteins that were differentially expressed with an average ratio of ± 5-fold after DeCyder software analysis of DIGE gels were considered. Two experiments were conducted with similar results.

### Protein identification by mass spectrometry

**Figure 4:**
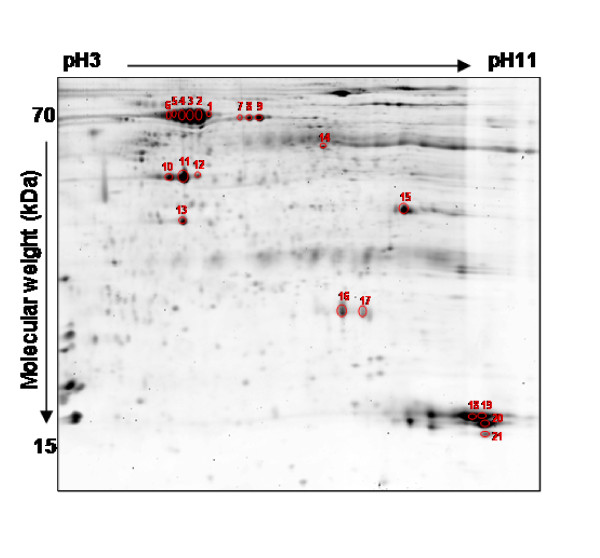
**Preparative 2-D gel of pooled proteins from *Rhipicephalus *spp.ticks**. Sixty four μg of total proteins from the mixture of infected and uninfected ticks were resolved by isoelectric focusing at pH 3-11 using IPG strips, followed by 12% SDS gel electrophoresis in the second dimension. Proteins of interest were analyzed by MS (circled and numbered on the figure).

A Sypro Ruby image of the preparative gel was developed for mass spectrometry analysis of differentially expressed proteins (Figure 4). After DeCyder analysis of the preparative gel, 21 spots with an average ratio of ± 5-fold and that appeared at least in two of the four groups under comparison were selected for MS identification.

Excised spots were trypsin-digested and analyzed by MALDI-TOF MS. Fourteen proteins were identified by peptide mass fingerprinting (PMF) (Table 3). The proteins that could not be identified by PMF were analyzed by LC-MS/MS and 6 new spots were identified (Table 3). Of the 21 spots analyzed, only two could not be identified by any of the MS techniques employed, probably due to the limitation in the quantity of sample used.

**Table 3:** *Rhipicephalus *spp. tick differentially expressed proteins identified by MALDI-TOF MS and LC-MS/MS after saturation 2-D DIGE analysis.

Spot **number**^a)^	**Accession number**^b)^	**Protein ID**^b) ^	**Mw/pI**^c)^	**Protein Score **^d)^	Number of matched peptides	Sequence coverage (%)	**Average -fold change **^e)^	Tick/Pathogen
1	3319897	Albumin (*Canis familiaris*)	67.8/5.3	123	11	19.0	+ 5.7	*R. sanguineus/E. canis*
							+ 7.8	*R. bursa/T. annulata*

2	3319897	Albumin (*Canis familiaris*)	67.8/5.3	127	11	29.6	+24.6	*R. sanguineus/E. canis*
							+17.5	*R. bursa/T. annulata*

3	3319897	Albumin (*Canis familiaris*)	67.8/5.3	125	13	30.1	+38.8	*R. sanguineus/E. canis*
							+16.9	*R. bursa/T. annulata*

4	3319897	Albumin (*Canis familiaris*)	67.8/5.3	85	9	22.2	+20.2	*R. sanguineusy/E. canis*
							+18.0	*R. bursa/T. annulata*

5	3319897	Albumin (*Canis familiaris*)	67.8/5.3	106	12	26.5	+12.8	*R. sanguineus/E. canis*
							+26.9	*R. bursa/T. annulata*

6	3319897	Albumin (*Canis familiaris*)	67.8/5.3	128	11	28.0	+ 9.8	*R. sanguineus/E. canis*
							+28.6	*R. bursa/T. annulata*

7	-	Not identified	-	-	-	-	-5.1	*R. bursa/T. annulata*
							-5.0	*R. turanicus/A. ovis*

8	5164373	Pre-pro serum albumin (*Ovis aries*)	69.2/5.8	IT	41	31.3	-5.5	*R. bursa/T. annulata*
							-5.3	*R. turanicus/A. ovis*

9	193085052	Albumin precursor (*Capra hircus*)	66.3/5.6	88	17	27.6	-5.2	*R. bursa/T. annulata*
							-5.1	*R. turanicus/A. ovis*

10	241157545	Actin, putative (*Ixodes scapularis*)	37.6/5.4	121	8	31.3	-7.9	*R. sanguineus/R. conorii*
							-5.6	*R. bursa/T. annulata*
							-5.1	*R. turanicus/A. ovis*

11	59894747	Actin (*Ixodes ricinus*)	41.5/5.6	121	11	42.0	- 5.5	*R. sanguineus/R. conorii*
							+6.6	*R. sanguineus/E. canis*
							- 5.9	*R. bursa/T. annulata*

12	-	Not identified	-	-	-	-	- 5.1	*R. sanguineus/R. conorii*
							- 6.3	*R. bursa/T. annulata*
							- 5.7	*R. turanicus/A. ovis*

13	258499	Haptoglobin heavy chain, HpH chain (*Canis familiaris*)	27.3/5.8	104	7	37.1	+36.7 +28.3 +12.4	*R. sanguineus/R. conorii* *R. sanguineus/E. canis* *R. bursa/T. annulata*

14	215497327	Enolase (*Ixodes scapularis*)	21.5/8.9	IT	4	18.1	- 8.2	*R. sanguineus/R. conorii*
							- 6.7	*R. bursa/T. annulata*
							- 5.5	*R. turanicus/A. ovis*

15	67083997	Guanine nucleotide-binding protein (*Ixodes scapularis*)	36.0/7.1	IT	2	9.4	-28.7 - 7.9 - 5.1	*R. sanguineus/R. conorii* *R. bursa/T. annulata* *R. turanicus/A. ovis*

16	157399341	Unknown larval protein (*Rhipicephalus annulatus)*	19.1/6.16	IT	3	17.5	-25.6 -7.3 +18.5	*R. sanguineus/R. conorii* *R. bursa/T. annulata* *R. turanicus/A. ovis*

17	157399341	Unknown larval protein (*Rhipicephalus annulatus)*	19.1/6.16	IT	5	11.1	- 12.4 +17.8	*R. bursa/T. annulata * *R. turanicus/A. ovis*

18	44887976	Full hemoglobin subunit beta (*Chrysocyon brachyurus*)	16.1/8.0	112	10	59.6	+38.6	*R. sanguineus/R. conorii*
							+ 9.5	*R. sanguineus/E. canis*
							+17.0	*R. bursa/T. annulata*

19 (mix)	116618139	Conjugal transfer protein, ATPase (*Leuconostoc mesenteroides*)	95.8/5.8	86	16	17.1	+62.4	*R. bursa/T. annulata*
	44887976	Full hemoglobin subunit beta (*Chrysocyon brachyurus*)	16.1/8.0	75	8	56.2	+13.1	*R. sanguineus/E. canis *
							+38.6	*R. sanguineus/R. conorii*

20	44888810	Full hemoglobin subunit alpha (*Canis familiaris*)	15.3/8.0	111	7	63.8	+66.7 + 7.0 +35.9	*R. sanguineus/R. conorii* *R. sanguineus/E. canis * *R. bursa/T. annulata*

21	44888810	Full hemoglobin subunit alpha (*Canis familiaris*)	15.3/8.0	IT	7	29.3	+41.5	*R. sanguineus/R. conorii*
							+30.7	*R. bursa/T. annulata*

^a) ^Spot numbers refer to the 2 D gel proteins of interest that were analyzed by mass spectrometry (Figure 3).

^b) ^Accession number and protein identity are listed according to the NCBInr database for the best match.

^c) ^Abbreviations: Mw, molecular weight (kDa); pI, isoelectrical point.

^d) ^Protein score is-10*Log(*P*), where *P *is the probability that the observed match is a random event, it is based on NCBInr database using MASCOT Peptide Mass Fingerprinting searching program. When IT appears, it means that these proteins were identified by LC-MS/MS.

^e) ^**+ **indicates an increase in protein expression and **- **indicates a decrease in protein expression in infected ticks.

Many of the identified spots corresponded to different isoforms to the same protein, which resulted in the identification of 9 unique proteins. Of these 9 proteins, 4 corresponded to host proteins (haptoglobin, albumin and alpha and beta hemoglobin) and the rest were assigned to tick or pathogen proteins (Table 3).

The host proteins identified are highly abundant plasma proteins. The higher levels of haptoglobin detected in all infected ticks (spot 13) could be explained by an increase in the circulating plasma levels of this stress/inflammatory acute-phase protein (APP) in the host caused by pathogen infection, a fact that has already been shown in bovine tropical theileriosis [30-32]. Albumin and alpha and beta hemoglobin levels varied between different tick species.

Increased haptoglobin, albumin and hemoglobin levels in questing *R. sanguineus *infected with *R. conorii *suggested that these proteins (or protein fragments) may be stored and transmitted intrastadially in infected ticks [16]. These results suggested that infection could modify tick digestion process, thus resulting in increased concentration of some plasma proteins ingested with blood meal in infected ticks. Additionally, proteins such hemoglobin fragments may have a role in tick immune defense mechanisms [33-36]. However, *T. annulata *infection could cause a reorganization of the different albumin isoforms without changes in the global quantity of the protein. More studies are needed in order to test these hypotheses.

Tick proteins differentially expressed in infected ticks included actin, enolase, guanine nucleotide-binding protein and an unknown larval protein (Table 3). Actin is a cytoskeleton component that appeared under-expressed in all infected *Rhipicephalus *spp. ticks except in *R. sanguineus *infected with *E. canis *were actin was over-expressed. *Rickettsia*, *Theileria*, *Ehrlichia *and *Anaplasma *infections cause rearrangements of actin cytoskeleton in mammalian [37-40] and invertebrate host cells [5,41]. The remodeling of the actin cytoskeleton by pathogen infection could explain the changes in actin detected in infected ticks. Differences in actin differential expression between different groups of infected ticks may be due to differences in pathogen infection and/or development mechanisms [5,42,43].

Host lipid metabolism including enolase function is affected in host-pathogen interactions [24,44-50]. Therefore, under-expression of enolase in infected *Rhipicephalus *spp. ticks could reflect changes in lipid metabolism induced by pathogen infection.

The other two tick proteins differentially expressed in response to infection, guanine nucleotide-binding protein and the unknown larval protein, are difficult to relate to infection and exhibited different expression profiles depending on the tick/pathogen tested. These differences could arise from both the tick and the pathogen. In this respect, *R. turanicus *infected with *A. ovis *showed some distinctive features in protein expression profiles when compared to other tick/pathogen tested (Figure 2 and 3; Table 2).

Although feeding ticks were incubated for three days in the laboratory to complete blood meal digestion prior to dissection and RNA/DNA/protein extraction, host proteins identified were highly abundant blood proteins that were probably masking proteins with lower abundance but perhaps more biologically relevant. Spot overlapping in 2-D electrophoresis is a problem described by different investigators [51-53]. In this work, pathogen proteins co-migrated with host haptoglobin and hemoglobins (spots 13, 18 and 19). For example, a bacterial ATPase was identified in spot 19 by peptide mass fingerprinting (table 3). Additionally, α-proteobacteria peptides IGYVSGMSGR (peptidase M2; GenBank accession number 220905421) and PIVYSAETQR (predicted protein; 227819201) were sequenced in spots 13 and 18, respectively, after *de novo *interpretation of non-assigned high-quality spectra.

Recently, Wickramasekara et al. [16] detected by MS host blood proteins in different tick species several months after feeding and molting. In our experiments, differences in protein profiles were found when comparing the two groups of uninfected *R. sanguineus *control ticks analyzed (Figure 2, left images), those suggesting that some of the tick differentially expressed proteins may be affected by tick feeding while other were masked by more abundant host proteins in feeding ticks. In future studies, it may be necessary to increase the time between tick collection and analysis to reduce the quantity of host blood proteins present in the tick.

### Analysis of mRNA levels for selected differentially expressed tick proteins

**Figure 5:**
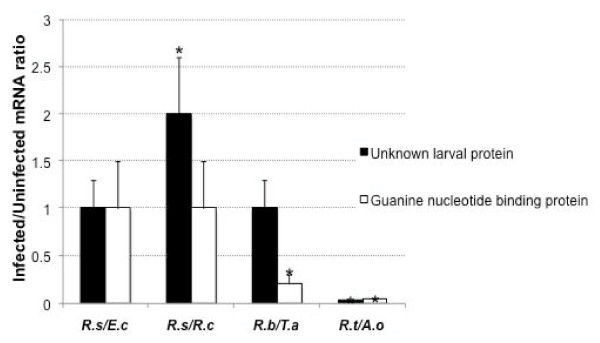
**mRNA levels of genes coding for selected differentially expressed proteins**. The genes encoding for differentially expressed unknown larval and guanine nucleotide-binding proteins were selected for mRNA analysis by real-time RT-PCR. mRNA levels were characterized in individual whole ticks naturally-infected with different pathogens using sequence-specific oligonucleotide primers. In all cases, matching groups of uninfected tick samples were analyzed concurrently for comparison. The graph depicts the infected to uninfected mRNA ratio (+SD) calculated by dividing normalized mRNA levels in infected ticks by the average of the normalized mRNA level in uninfected control ticks. Normalized mRNA levels were compared between infected and uninfected ticks by Student's t-Test (*P < 0.05). Abbreviations: *R.s/E.c*, *R. sanguineus/E. canis*; *R.s/R.c*, *R. sanguineus/R. conorii*; *R.b/T.a*, *R. bursa/T. annulata*; *R.t/A.o*, *R. turanicus/A. ovis*.

To correlate differential expression of tick guanine nucleotide-binding and unknown larval protein mRNA levels, the RNAs from individual infected and uninfected ticks were analyzed by real-time RT-PCR (Figure 5). The results showed at the mRNA level differences in differential protein expression between tick/pathogen groups (Table 4). In most cases mRNA and protein levels were unchanged or down-regulated, differences that were regarded as not significant due to the lack of statistical analysis in proteomics data. In most cases, a good correlation was obtained between mRNA and protein levels, thus suggesting that gene expression was regulated at the transcriptional level. However, for the unknown larval protein opposite results were obtained for *R. sanguineus*/*R. conorii *and *R. turanicus*/*A. ovis *(Table 4). These differences suggested that the unknown larval protein levels may be regulated at different levels in different *Rhipicephalus *spp. and/or in response to different pathogens infection. Additionally, these results suggested that at least in some cases the expression of the unknown larval protein might be regulated at the post-transcriptional level. These results reinforced the importance of conducting proteomics and transcriptomics studies together to fully characterize host response to infection [5,25].

**Table 4:** Comparative analysis between protein and mRNA levels for selected differentially expressed tick proteins.

		Infected/Uninfected	
		
Tick protein	Tick/pathogen	Protein	mRNA
Unknown larval protein			

	*R. sanguineus/E. canis*	Down	Up

	*R. sanguineus/R. conorii*	Unchanged	Unchanged

	*R. bursa/T. annulata*	Down	Unchanged

	*R. turanicus/A. ovis*	Up	Down

Guanine nucleotide-binding protein			

	*R. sanguineus/R. conorii*	Down	Unchanged

	*R. sanguineus/E. canis*	Unchanged	Unchanged

	*R. bursa/T. annulata*	Down	Down

	*R. turanicus/A. ovis*	Down	Down

The mRNA levels of the differentially expressed tick guanine nucleotide-binding and unknown larval proteins were analyzed by real-time RT-PCR with RNAs from individual infected and uninfected ticks and correlated with protein levels determined by 2-D DIGE.

### Technical considerations

Herein tick proteins were extracted with TriReagent (Sigma) to allow for simultaneous extraction and analysis of DNA, RNA and proteins from the same sample. This procedure resulted in low protein amounts which could have been improved by using more suitable methods employed for 2-D analysis [54-56]. However, DNA and RNA were required to determine pathogen infection and to characterize mRNA levels for some of the differentially expressed proteins, respectively. Moreover, after analysis of 300 collected ticks, only 16 were infected with a pathogen, and in some cases experimental groups positive for the same pathogen contained only 2 ticks. These facts made the amount of proteins available for analysis the principal limitation in the development of this study and even using a highly sensitive technique such as saturation DIGE labeling, it was necessary to mix protein samples from different individuals of the same tick species to carry out the experiments. Protein mixing was not required for the saturation labeling, but to run a preparative gel for identification of differentially expressed proteins by mass spectrometry.

Under some circumstances with naturally (and not experimentally) infected ticks, it may be difficult to obtain a large number of infected ticks in order to perform the protein isolation with optimized methods for further proteomics analysis independently of DNA and RNA extractions, which would improve the quantity and quality of protein samples. Using optimized methods for protein extraction would allow analysis of individual samples and thus increase the statistical power of results. Another important issue to be considered for further studies is the high proportion of host proteins that were identified. Whereas this work focused on differentially expressed tick proteins, to improve the sensitivity of proteomics analysis in feeding ticks and detect proteins with low abundance it would be useful to reduce the concentration of highly-abundant host proteins before protein profiling by using different commercial strategies developed for the depletion of these proteins in different complex biological samples [57-59].

## Conclusions

The results reported here proved that saturation DIGE technology in combination with MS analysis is a powerful tool for the study of host-tick-pathogen interactions using a small number of ticks. Herein, host, tick and pathogen proteins were identified and shown to be present in different amounts in infected and uninfected ticks. These results supported that pathogen infection affect tick protein expression. However, these results should be considered preliminary due to the limitations imposed by technical consideration discussed above. Therefore, more comprehensive analyses are required with optimized protein extraction methods to allow analysis of individual tick samples and by removing most abundant host proteins from tick samples in an attempt to identify a larger number of tick differentially expressed proteins through multi-dimensional LC-MS/MS and correlating transcriptomics and proteomics data to characterize the regulatory networks at the tick-pathogen interface.

## Competing interests

The authors declared that they have no competing interests.

## Authors' contributions

MV and JF conceived and designed the experiments. JF, MV, AT, SC and JV coordinated the experiments. MV, AT, YN, ZZ, AM, AA, SS and GLB performed the experiments. MV, YN, AM, and JF analyzed the data. MV and JF drafted the manuscript. All authors read and approved the final manuscript.
